# CD83 antigen expression and its role in the progression of systemic malignancies

**DOI:** 10.1590/S1516-31802013000100027

**Published:** 2013-04-01

**Authors:** Shailendra Kapoor

**Affiliations:** I MD. Private practice, Mechanicsville, Virginia, United States.

I read with great interest the recent article by Borges et al.^1^ CD83 may play a major role in the progression of systemic tumors other than fibroadenomas. Shorter survival rates are seen among patients with gastric carcinomas that demonstrate a lesser number of CD83-positive dendritic cells within the tumor.^2^ In fact, the prognosis and clinical outcome in cases of gastric carcinoma is inversely related to the density of CD83-positive dendritic cells.^3^ Similarly, in cases of gallbladder carcinoma, the tumor prognosis is influenced by infiltration of CD83-positive dendritic cells. For instance, in a recent study, Furihata et al. determined the CD83 index in gallbladder carcinomas by using the formula CD83 (+) DCs/ (CD83 (+) DCs plus CD1a (+) DCs, where DC stands for dendritic cell.^4^ They showed that individuals with a higher CD83 index (greater than 0.316) had a better overall prognosis and clinical outcome.

In females with breast carcinomas, an inverse relationship exists between lymph node metastasis and CD83-positive dendritic cells. In fact, those who demonstrate higher numbers of CD83-positive dendritic cells and also exhibit lymph node metastasis have better survival rates than shown by those with smaller numbers of CD83-positive cells.^5^ Longer “relapse-free survival” is also seen among individuals with higher CD83 dendritic cell levels. Similarly, in cases of cervical carcinoma, higher incidence of CD83 polymorphisms has been noted. Females with SNP rs9370729 on CD83 are more likely to develop cervical adenocarcinomas. A similar relationship is seen between cervical adenocarcinomas and SNP rs75074 on CD83. On the other hand, females with the CC genotype of SNP rs750749 on CD83 demonstrate almost half the risk of developing cervical malignancies, in contrast with those who exhibit the TT genotype.^6^

Higher levels of “soluble CD83” are seen in certain cases of chronic lymphocytic leukemia. Disease progression in cases of chronic lymphocytic leukemia may be strongly influenced and regulated by “soluble CD83”.^7^ Recently, CD83 monoclonal antibodies were developed from 9D8 hybridoma cell lines, and this may prove to be of great benefit in oncology.^8^

The above examples clearly illustrate the significance of assessing CD83 status in the above tumors and the need for further human studies in this regard.
