# Supplementary material for: CD83 antigen expression and its role in the progression of systemic malignancies
Source: Sao Paulo Med J. 2013 Apr 1;131(2):137–8. doi: 10.1590/S1516-31802013000100027 (PMC10871722; doi:10.1590/S1516-31802013000100027)
Supplement: Related texts

# Reply: Let’s be attentive to the CD83 antigen expression in dendritic cell!

**DOI:** 10.1590/S1516-31802013000100027

**Authors:** Marcus Nascimento Borges, Afonso Celso Pinto Nazário

**Affiliations:** II MD. Gynecologist and Mastologist, Department of Gynecology, Universidade Federal de São Paulo - Escola Paulista de Medicina (Unifesp-EPM), São Paulo, Brazil.; III MD, PhD. Full Professor, Department of Gynecology, Universidade Federal de São Paulo - Escola Paulista de Medicina (Unifesp-EPM), São Paulo, Brazil.

We read the letter to the editor “CD83 antigen expression and its role in the progression of systemic malignancies”, that was written by Dr. Shailendra Kapoor, with great attention and interest. It shows that CD83 antigen expression in dendritic cells should represent a clinical marker for progression and prognosis in some malignant neoplasms like gastric carcinomas and gallbladder carcinomas. The letter also mentions that positive CD83 antigen expression in dendritic cells is inversely related to lymph node metastasis in women with breast carcinomas.

We are grateful to Dr. Kapoor for referring to our article in his letter. In fact, this was the first study on this issue in the Department of Gynecology, Unifesp, and has led us to great interest in CD83 antigen expression and its relationship with breast neoplasms.

Currently, new research is underway, with the aim of showing CD83 antigen expression and its significance in cases of other breast neoplasms. We consider that positive CD83 antigen expression in dendritic cells may be a biological marker relating to clinical tumor progression and may also have an important role in the immunological response against breast neoplasms.

We totally agree with Dr. Kapoor that this issue deserves careful study in order to improve our knowledge and, perhaps, open our minds to new thinking in relation to the progression and treatment of neoplasms.
